# Demographic Responses to Oxidative Stress and Inflammation in the Wandering Albatross (*Diomedea exulans*)

**DOI:** 10.1371/journal.pone.0133967

**Published:** 2015-08-14

**Authors:** David Costantini, Aurelie Goutte, Christophe Barbraud, Bruno Faivre, Gabriele Sorci, Henri Weimerskirch, Karine Delord, Olivier Chastel

**Affiliations:** 1 Department of Biology, University of Antwerp, Universiteitsplein 1, 2610, Wilrijk, Belgium; 2 IBAHCM, University of Glasgow, Glasgow, United Kingdom; 3 Centre d’Etudes Biologiques de Chizé (CEBC), UMR7372- CNRS/Univ. La Rochelle, F-79360, France; 4 École Pratique des Hautes Études (EPHE), UPMC Univ. Paris 06, UMR 7619-CNRS, 4 pl. Jussieu, F-75005 Paris, France; 5 Université de Bourgogne, UMR–CNRS 6282 Biogéosciences, 6 Boulevard Gabriel, 21000, Dijon, France; Universidad de Granada, SPAIN

## Abstract

One of the major challenges in ecological research is the elucidation of physiological mechanisms that underlie the demographic traits of wild animals. We have assessed whether a marker of plasma oxidative stress (TBARS) and plasma haptoglobin (protein of the acute inflammatory phase response) measured at time t predict five demographic parameters (survival rate, return rate to the breeding colony, breeding probability, hatching and fledging success) in sexually mature wandering albatrosses over the next four years (*Diomedea exulans*) using a five-year individual-based dataset. Non-breeder males, but not females, having higher TBARS at time t had reduced future breeding probabilities; haptoglobin was not related to breeding probability. Neither TBARS nor haptoglobin predicted future hatching or fledging success. Haptoglobin had a marginally positive effect on female survival rate, while TBARS had a marginally negative effect on return rate. Our findings do not support the role for oxidative stress as a constraint of future reproductive success in the albatross. However, our data point to a potential mechanism underlying some aspects of reproductive senescence and survival. Our results also highlight that the study of the consequences of oxidative stress should consider the life-cycle stage of an individual and its reproductive history.

## Introduction

A key question in ecology concerns the physiological mechanisms that determine demographic traits of natural animal populations. One physiological mechanism thought to be important in this regard is oxidative stress, caused by oxidative damage to biomolecules, because it may reduce growth, fertility or survival [1]. There is, therefore, a clear necessity for individuals to manage oxidative stress in an efficient manner to optimize trade-offs among traits, such as reproductive investment and survival [1]. For instance, it is expected that high investment in reproduction would result in high oxidative stress, which would come at a cost of decreasing survival due to its damaging effects.

Progressive damage to biomolecules can also be associated with an inflammatory response, a mechanism used by the organism to protect itself from a stressful agent [2]. Inflammation-inducible proteins, such as haptoglobin, can limit the spread of oxidative stress across tissues by binding molecules with pro-oxidant activity [3]. It is, therefore, relevant to assess the levels of oxidative stress and the levels of inflammation-inducible proteins (i) to elucidate mechanisms underlying individual variation in survival and in reproductive success and reproductive lifespan and (ii) to identify markers that are related to fitness traits and may therefore be used to assess and predict demographic traits of a population.

Field studies that have assessed the relationship between oxidative status and survival or reproduction in vertebrates have generated contrasting results. Individuals having higher antioxidant defences (i.e., molecules that protect cells against oxidative damage) have been described as being more likely to return to the breeding ground the next year when compared to individuals having lower antioxidant defences [4–5]. However, other studies did not find any relationship between oxidative stress and survival proxies [6–7]. Further work has also shown that individuals with lower oxidative stress had higher reproductive success [1, 7–8]. What is lacking are studies that track the survival and reproductive history of free-living individuals over multiple years and relate these demographic variations to markers of oxidative status.

Here, we have assessed whether a marker of plasma oxidative stress (thiobarbituric acid reactive substances, TBARS) and plasma haptoglobin measured at time t predict a number of demographic parameters (survival rate, return rate to the breeding colony, breeding probability, hatching and fledging success) in the next four years in the wandering albatross (*Diomedea exulans*) using a longitudinal individual-based dataset (5 years). TBARS and haptoglobin are positively correlated in our study species (see results). In a previous cross-sectional study on this albatross species, we also found that TBARS were higher in older non-breeding individuals and in breeding birds, while haptoglobin was lower in breeding males than breeding females and both non-breeding males and females [9]. Hence, we expected to find (i) relationships between TBARS and survival or reproductive traits and (ii) that any relationships between haptoglobin and demographic traits to differ between sexes.

## Materials and Methods

### Study area, species and field study

Field procedures and blood sampling were authorized by the Ethics Committee of French Polar Institute and by the Comité de l’Environnement Polaire. The field study was carried out on Possession Island in the southwestern Indian Ocean (46.8°S, 51.8°E), where 300–400 pairs of wandering albatrosses breed each year. Wandering albatrosses return to their breeding grounds in December and females lay a single egg in late December–early January. Both parents incubate alternatively until hatching in March. All birds had been ringed as part of a long-term capture-mark-recapture program [10], with nestlings being ringed since 1965. From 21 December 2007 to 04 March 2008, we captured 144 sexually mature wandering albatrosses (59 male breeders; 50 female breeders; 31 male non-breeders; 4 female non-breeders) on the breeding grounds and a sample of venous blood was taken from the tarsus within 3 minutes of capture with a 1-mL heparinized syringe and a 25-gauge needle. The volume of blood drawn never exceeded 0.05% of the bird’s body mass (8–12 Kg). The blood was centrifuged to separate the plasma (for oxidative damage and inflammation analyses) from the red blood cells (for molecular sexing), which were then stored at -20°C prior to laboratory analyses. We also scored the density of ticks *Ixodes uriae* found on the head plumage during a 10 minutes restraint in order to test whether haptoglobin reflects an inflammatory response induced by these parasites. Scores ranged from 0 (no parasites) to 4 (very high density of ticks). The colony of albatrosses was then subsequently visited each next year up until 2012. During this period, we collected longitudinal data on survival and return rate, breeding probability, hatching and fledging success.

### Molecular sexing

Molecular sexing was determined on red blood cells at the Centre d’Etudes Biologiques de Chizé (CEBC), by polymerase chain reaction (PCR) amplification of part of two highly conserved genes (CHD) present on the sex chromosomes, as detailed in [11].

### Measurement of plasma oxidative stress

The Cayman's TBARS assay (Cayman Chemical Company, Ann Arbor USA) was used to measure the thiobarbituric acid reactive substances in plasma. This method provides a general quantification of oxidative damage molecules that occur in the plasma, such as those generated by lipid peroxidation or carbonylation. The principle of the assay is based on the formation of an adduct between the thiobarbituric acid and the oxidative damage molecules under high temperature (90–100°C) and acidic conditions, which generates a colour directly proportional to the concentration of oxidative damage molecules. First, 10 μl of each plasma sample or standard were added to 10 μl of sodium dodecyl sulphate into 500 μl vials, and mixed. Then 400 μl of colour reagent (132.5 mg of thiobarbituric acid diluted into 12.5 ml of an acetic acid solution and 12.5 ml of a sodium hydroxide solution) were added to each plasma solution, and caped vials were kept in boiling water for one hour. After one hour the vials were removed from the boiling water and immediately put onto ice for 10 minutes in order to stop the reaction. Finally, 150μl of each solution was randomly pipetted in well plates and readings were taken at 530 nm. Standard curves were obtained from serial dilutions of an standard of MDA (from 0 to 50μM). The coefficient of variation of sample measures was 9.0%.

### Measurement of haptoglobin

Plasma haptoglobin (inflammation-inducible protein) was measured using a colorimetric assay (Tri-Delta Development, Ireland) based on hemoglobin-binding reaction. In plasma, haptoglobin binds free hemoglobin released from erythrocytes, so inhibiting its pro-oxidative activity. First, 7.5 μl of each plasma sample or standard were randomly pipetted in well plates. To each well, 100 μl of a solution of hemoglobin and 140 μl of a solution of chromogen respectively were added. Plates were then agitated and left to incubate for 5 minutes at room temperature. Then solutions were read at 630 nm. Standard curves were obtained from serial dilutions of an initial standard (0 to 2.5 mg/ml). The coefficient of variation of sample measures was 3.7%.

### Statistical analyses

Data on TBARS and haptoglobin were previously published by our group [9, 12]. Here, we are revisiting this data-base in order to analyse the effects of both TBARS and haptoglobin on demographic traits using the capture-recapture data of sampled individuals from 2008 to 2012. We used multi-state mark-recapture models (MSMR) as developed by [13–14]. This model includes eight states: dead, failed breeder on egg (FBE, defined as an individual that was observed with one egg that failed to hatch), failed breeder on chick (FBC, defined as an individual that was observed with one chick but that failed to fledge the chick), successful breeder (SB, defined as an individual that fledged one chick), observable non-breeder (ONB, defined as an individual that was observed at the colony but that was not observed with an egg or a chick), and three unobservable states consisting of non-breeders that were observed at the colony during the previous breeding attempt (PONB), non-breeders whose previous breeding attempt failed (PFB) and non-breeders whose previous breeding attempt was successful (PSB). The state dead (†) was an absorbing state representing death or permanent emigration from the study areas. The unobservable states account for temporary absence corresponding to birds that skip breeding after breeding unsuccessfully or successfully.

States occupied are not directly observed; rather at each occasion t, an event happens and is recorded leading to an observed encounter history. In our case, we considered five events; 0 = ‘‘not observed”, 1 = ‘‘seen as a failed breeder on egg”, 2 = ‘‘seen as a failed breeder on chick”, 3 = “seen as a successful breeder”, 4 = ‘‘seen as a non-breeder”, which were used to establish capture histories. Events and states are considered as random variables, and it is assumed that an event at occasion t depends only on an underlying state (which is not observed) of the individual at the moment, and that successive states obey a Markov chain. Models were parameterized in terms of the probability of survival (*s*), the probability to return at the colony given survival (*r*), the probability of breeding given return at the colony (*β*), the probability of successful hatching given breeding (*ω*), the probability of successful fledgling given hatching (*γ*), and the detection probability (*p*) (Table 1). Transition probabilities between states were modelled with a five-step procedure where *s*, *r*, *β*, *ω* and *γ* were considered as five successive steps in transition matrices. We chose a MSMR approach as this enables us to take into account the probability of detecting individuals upon their return to the study site. It also enables us to take into account the previous breeding state of individuals, which may be important to obtain unbiased estimates of demographic parameters [15].

**Table 1:** Definition of parameters used in the multistate mark–recapture model.

Parameter	Definition
*s* ^*t*^_*s*_	Probability that an individual in state *s* at time *t* survives to time *t + 1* and does not permanently emigrate from the study area
*r* ^*t*^_*s*_	Probability that an individual in state *s* at time *t* returns at the colony to time *t + 1* given that it survives to *t + 1*
*β* ^*t*^_*s*_	Probability that an individual in state *s* at time *t* breeds at time *t + 1* given that it survives to and returns at the colony at time *t + 1*
*γ* ^*t*^_*s*_	Probability that an individual in state *s* at time *t* incubates successfully at time *t + 1* given that it survives to, returns at the colony and breeds at time *t + 1*
*δ* ^*t*^_*s*_	Probability that an individual in state *s* at time *t* raises successfully one chick at time *t + 1* given that it survives to, returns at the colony and incubates successfully at time *t + 1*
*p* ^*t*^_*s*_	Probability that an individual in state *s* at time t is encountered at time *t + 1*

Several constraints were made to ensure that the parameters of the model were estimable. The state “dead” being explicitly included in the model but being never encountered, initial encounter probability was fixed to 0, transition probabilities from the state “dead” to the other states were fixed to 0 and capture probability was fixed to 0 [16–17]. The probability of seeing individuals in unobservable states and transitions between unobservable states were constrained to 0. Since some individuals were observed breeding in the year consecutive to a successful breeding event [18], we did not constrain *β*_*SB*_ to 0. To limit redundancy in survival parameters, we did not consider models where survival probabilities all varied separately [19]. Because our capture-recapture analyses relied on a limited number of individual capture histories, a limited number of recapture occasions and a relatively large number of unobservable states we constrained i) parameters *s*, *r*, *β*, *ω*, *γ* and *p* to be constant over time, ii) return rates to be similar for ONB, PFB, PSB, and PONB, and iii) breeding probabilities to be similar for PFB, PSB and PONB. With these constraints the initial model was full-rank. Note that we ran a model where all demographic parameters were time and state dependent but this model was highly rank deficient.This MSMR model was parameterized by the survival–transition probabilities matrix shown in Table 2.

**Table 2:** Multi-state mark-recapture models were parameterized by the survival–transition probabilities matrix.

	FBE	FBC	SB	ONB	PFB	PSB	PONB	†
FBE	srβ(1-ω)	srβω(1-γ)	srβωγ	sr(1-β)	s(1-r)	―	―	*
FBC	srβ(1-ω)	srβω(1-γ)	srβωγ	sr(1-β)	s(1-r)	―	―	*
SB	srβ(1-ω)	srβω(1-γ)	srβωγ	sr(1-β)	―	s(1-r)	―	*
ONB	srβ(1-ω)	srβω(1-γ)	srβωγ	sr(1-β)	―	―	s(1-r)	*
PFB	srβ(1-ω)	srβω(1-γ)	srβωγ	sr(1-β)	―	―	―	*
PSB	srβ(1-ω)	srβω(1-γ)	Srβωγ	sr(1-β)	―	―	―	*
PONB	srβ(1-ω)	srβω(1-γ)	srβωγ	sr(1-β)	―	―	―	*
†	―	―	―	―	―	―	―	*

† indicates dead

* indicates the complementary parameter (complement of the sum of positive row entries)

Because we were interested in testing for sex-specific effects of TBARS and haptoglobin on demographic parameters, we started from an initial model including an effect of sex on each parameter. Hence, in our initial model survival was sex dependent, return probability was sex and state dependent with 4 states (FBE, FBC, SB, and NB where NB was a global state including the ONB, PONB, PFB and PSB states), breeding probability was sex and state dependent with 5 states (FBE, FBC, SB, ONB, and UNB where UNB was a global state including the PONB, PFB and PSB states), hatching and fledging probabilities were sex and state dependent with 7 states (FBE, FBC, SB, ONB, PFB, PSB, and PONB), and detection probability was sex and state dependent with 4 states (FBE, FBC, SB, ONB). We tested for sex-differences and state-dependency for each parameter.

Then, we tested for an effect of TBARS and haptoglobin on demographic parameters to test the hypothesis that their levels in one breeding season may influence the long-term survival and breeding outputs of an individual over the following four years. We built MSMR models where each demographic parameter *θ* was modeled as a function of an individual covariate *C* using a logit link function: logit(*θ*) = *a*+*b*×*C*_*i*_, where *a* is an intercept, *b* is a slope and *C*_*i*_ is the covariate for individual i. When *b* < 0, or *b* > 0, the covariate *C* has a negative or positive effect on the demographic parameter *θ*, respectively. The covariate *C* is the standardized value of TBARS or haptoglobin. The effect of the covariate *C* was first tested on each demographic parameter and for each different state. Because breeding probability differed between males and females and between the previous states of the individuals and because we only sampled 4 female non-breeders, we did not test the effects of TBARS or haptoglobin on breeding probability of females previously in the state non-breeders. We used the 95% confidence interval of the slope parameters and Akaike’s Information Criterion corrected for small sample size (AICc [20]) for inference, taking into account the minimum recommended AICc difference of 2.

We tested the goodness-of-fit (GOF) of the time-dependent MSMR model using U-CARE [21]. Model selection was based on AICc and all models were run under program E-SURGE 1.8.5 enabling us to split transition probabilities between states [17]. To avoid estimating parameters at a local minimum of the likelihood function, each model was run 5 times with random initial values.

## Results

TBARS and haptoglobin were positively correlated (*r* = 0.145, *p* = 0.041), (sampling date was not correlated with TBARS nor with haptoglobin (*p*≥0.14), ectoparasite density in the plumage (scores from 0 to 4) was not correlated with TBARS (*r* = 0.06, *p* = 0.31), but was slightly positively correlated with haptoglobin (*r* = 0.203, *p* = 0.037).

The GOF tests are reported in Table 3. The best model according to AICc (model #21, Table 4) suggested that males had a lower survival probability and breeding probability than females, but males and females did not differ in return rate, hatching success and fledgling success. Return rates were lower in individuals that were in states SB in the preceding year than individuals that were in states failed breeder (FB = FBE and FBC) and non-breeder (ONB, PFB, PSB and PONB). Breeding probability was the lowest in individuals that were previously in states SB, higher in individuals that were in states ONB, then in states FB, and the highest in unobservable non-breeders (UNB). Hatching success and fledgling success were lower in individuals that were in states breeders (FB and SB) than in non-breeders.

**Table 3:** GOF results of all tests. 3G.SR and M.LTEC could not be calculated because of the low number of data.

Test	Male	Female
WBMA (memory)	χ^2^ = 4.125, df = 7, p = 0.765	χ^2^ = 3.083, df = 7, p = 0.877
3G.SR (transience)	Not calculated	Not calculated
3G.Sm (composite test)	χ^2^ = 5.518, df = 12, p = 0.938	χ^2^ = 0, df = 10, p = 1
Test 3G	χ^2^ = 9.643, df = 19, p = 0.961	χ^2^ = 3.083, df = 17, p = 1.000
M.ITEC (immediate trap-dependance)	χ^2^ = 12.065, df = 2, p = 0.002	χ^2^ = 8.126, df = 2, p = 0.017
M.LTEC (long-term trap-dependance)	Not calculated	Not calculated
Test M	χ^2^ = 12.065, df = 2, p = 0.002	χ^2^ = 8.126, df = 2, p = 0.017
Total	χ^2^ = 21.708, df = 21, p = 0.417	χ^2^ = 11.209, df = 19, p = 0.917

**Table 4:** Effects of sex and states on detection probability (p), probabilities of adult survival (s), breeding (β), hatching (γ), and fledgling (δ).

**Testing for the effects of sex on *s*, *r*, *β*, *ω*, *γ*, and *p***	#	Rank	Deviance	ΔAICc
Sex-differences in *s* and *β*	7	31	1628.94	0
Sex-differences in s, *β* and *γ*	6	35	1624.95	5.24
Sex-differences in s, *β*, *ω* and *γ*	4	39	1624.90	14.57
Sex-differences in r, *β*, *ω* and *γ*	3	42	1626.13	22.95
Sex-differences in *s*, *r*, *β*, *ω* and *γ*	2	43	1622.89	22.12
Sex-differences in *s*, *r*, *β*, *ω*, *γ*, and *p*	1	47	1618.47	27.42
Sex-differences in s, *ω* and *γ*	5	34	1666.45	44.42
**Testing for the effects of states on *p***	#	Rank	Deviance	ΔAICc
Differences among (FBE FBC), SB and ONB	8	30	1629.69	0
Differences among FBE, FBC, SB and ONB	7	31	1628.94	1.53
Similitude among FBE, FBD, SB and ONB	9	28	1667.71	33.49
**Testing for the effects of states on r**	#	Rank	Deviance	ΔAICc
Differences among (FBE FBC) SB, and (ONB PFB PSB PONB)	10	29	1629.97	0
Differences among FBE,FBC,SB, and (ONB PFB PSB PONB)	8	30	1629.69	1.99
Differences among (FBE FBC SB) and (ONB PFB PSB PONB)	11	28	1691.79	59.56
Similitudes among FBE FBC SB ONB PFB PSB PONB	12	27	1737.14	102.66
**Testing for the effects of states on β**	#	Rank	Deviance	ΔAICc
Differences among (FBE FBC), SB, ONB, and (PFB PSB PONB)	13	27	1634.18	0
Differences among FBE, FBC, SB, ONB, and (PFB PSB PONB)	10	29	1629.97	0.30
Differences among (FBE, FBC, SB), ONB, and (PFB PSB PONB)	14	25	1673.25	34.60
Differences among (FBE FBC), SB, and (ONB PFB PSB PONB)	15	25	1713.04	74.39
Similitudes among FBE, FBC, SB, ONB, PFB, PSB, PONB	16	21	1733.53	86.05
**Testing for the effects of states on *ω***	#	Rank	Deviance	ΔAICc
Differences between (FB SB) and (ONB UNB)	18	25	1636.16	0
Differences among FB, SB and (ONB UNB)	17	26	1635.98	2.06
Differences among FB, SB, ONB, UNB	13	27	1634.18	2.49
Similitudes among FB, SB, ONB, UNB	19	24	1651.54	13.16
**Testing for the effects of states on γ**	#	Rank	Deviance	ΔAICc
Differences between (FB SB) and (ONB UNB)	21	23	1636.55	0
Differences among FB, SB and (ONB UNB)	20	24	1636.30	1.96
Differences among FB, SB, ONB, UNB	18	25	1636.16	4.04
Similitudes among FB, SB, ONB, UNB	22	22	1651.45	12.70

**Fig 1:**
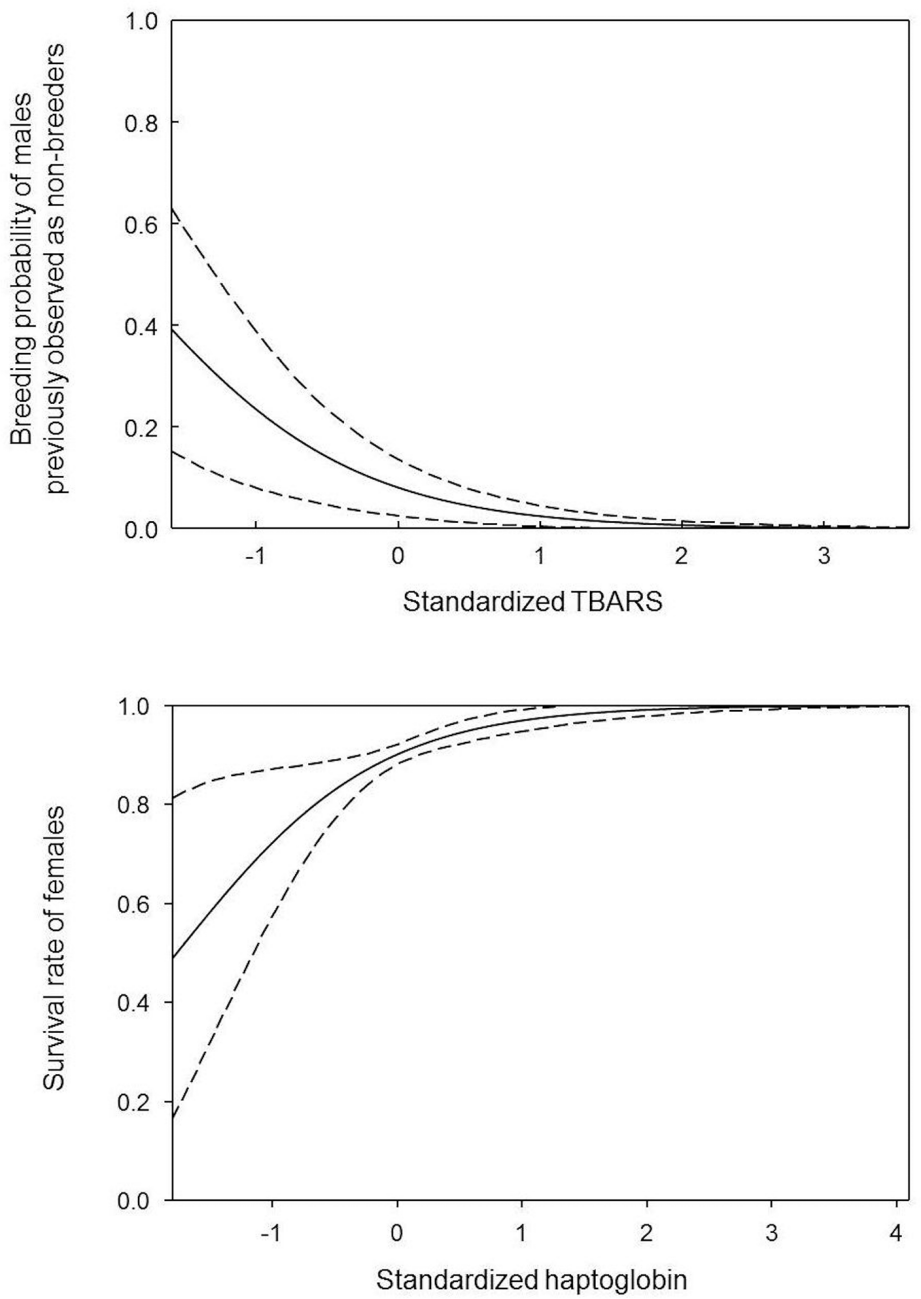
(a) Effect of TBARS on the breeding probability of males previously observed as non-breeders and (b) effect of haptoglobin on the survival rate of females. Dotted lines represent 95% confidence intervals estimated using the delta method [33].

Models 4, 9, 10, and 13 on the relationship between TBARS and demographic parameters had lower AICc than the intercept-only model 0 (Table 5A). However, the confidence intervals of slope parameter values for models 4 and 9 included 0 and the slope parameter value estimated in model 13 was aberrant with an extremely high value (Table 5A). The long-term breeding probability of males previously observed as non-breeders, but not that of females, was negatively related to TBARS (model 10, Table 5A, Fig 1A). Apart from a marginally negative effect of TBARS on return rate, TBARS did not predict any other reproductive or survival traits.

**Table 5:** Effects of TBARS (A) and haptoglobin (B) on demographic parameters (144 individuals). The estimated slope and 95% confidence intervals (CI) for models with a lower AICc than the intercept model (model 0) are given. FBE, individual that was observed with one egg that failed to hatch; FBC, individual that was observed with one chick that failed to fledge; SB, individual that fledged one chick; ONB, individual that was observed at the colony but that was not observed with an egg or a chick; non-breeders (i) that were observed at the colony during the previous breeding attempt (PONB), (ii) whose previous breeding attempt failed (PFB), (iii) whose previous breeding attempt was successful (PSB). * Although this model was full rank, the large value of the slope estimate is suspect and could be due to a low number of females transiting through the unobservable states.

Hypothesis	Model	Rank	Deviance	Δ AICc	Slope	CI-	CI+
**a. Effect of plasma oxidative damage (TBARS) on:**							
Breeding probability at year t+1 of males in state ONB at t	10	24	1380.534	0	-1.245	-2.053	-0.438
Breeding probability at year t+1 of females in state PFB, PSB or PONB at t	12	24	1386.838	6.304	29.651	26.853	32.449
Return rate at year t+1 of individuals in state SB at t	4	24	1388.305	7.771	-0.472	-0.952	0.008
Breeding probability at year t+1 of females in state SB at t	9	24	1389.666	9.132	1.243	-0.675	3.160
No effect on demographic parameters	0	23	1392.733	9.946			
Return rate at year t+1 of individuals in state ONB, PFB, PSB or PONB at t	5	24	1390.905	10.371			
Breeding probability at year t+1 of females in state FBE or FBC at t	7	24	1390.991	10.457			
Breeding probability at year t+1 of males in state PFB, PSB or PONB at t	11	24	1391.556	11.022			
Survival rate of males	1	24	1391.660	11.126			
Breeding probability at year t+1 of males in state FBE or FBC at t	6	24	1391.814	11.281			
Fledgling success at year t+1 of individuals in state ONB or UNB at t	16	24	1392.514	11.980			
Fledgling success at year t+1 of individuals in state FB and SB at t	15	24	1392.567	12.033			
Survival rate of females	2	24	1392.571	12.037			
Breeding probability at year t+1 of males in state SB at t	8	24	1392.591	12.057			
Hatching success at year t+1 of individuals in state ONB or UNB at t	14	24	1392.701	12.167			
Hatching success at year t+1 of individuals in state FB and SB at t	13	24	1392.701	12.167			
Return rate at year t+1 of individuals in state FBE or FBC at t	3	24	1392.733	12.199			
**b. Effect of haptoglobin on:**							
Survival rate of females	2	24	1388.919	0.000	1.251	-0.137	2.638
Return rate at year t+1 of individuals in state SB at t	4	24	1388.971	0.052	-0.472	-0.989	0.045
No effect on demographic parameters	0	23	1392.733	1.561			
Fledgling success at year t+1 of individuals in state ONB or UNB at t	16	24	1390.728	1.809			
Fledgling success at year t+1 of individuals in state FB and SB at t	15	24	1390.738	1.818			
Breeding probability at year t+1 of females in state PFB. PSB or PONB at t	12	24	1391.350	2.431			
Hatching success at year t+1 of individuals in state ONB or UNB at t	15	24	1391.405	2.486			
Breeding probability at year t+1 of females in state SB at t	9	24	1391.965	3.046			
Survival rate of males	1	24	1392.015	3.096			
Breeding probability at year t+1 of males in state ONB at t	10	24	1392.019	3.100			
Breeding probability at year t+1 of males in state PFB, PSB or PONB at t	11	24	1392.297	3.378			
Breeding probability at year t+1 of males in state FBE or FBC at t	6	24	1392.422	3.503			
Return rate at year t+1 of individuals in state FBE or FBC at t	3	24	1392.538	3.619			
Breeding probability at year t+1 of females in state FBE or FBC at t	7	24	1392.665	3.745			
Hatching success at year t+1 of individuals in state FB and SB at t	13	24	1392.711	3.791			
Breeding probability at year t+1 of males in state SB at t	8	24	1392.733	3.813			
Return rate at year t+1 of individuals in state ONB, PFB, PSB or PONB at t	5	24	1392.733	3.814			

ΔAICc values of the best models (models 2 and 4, Table 5B) for haptoglobin were less than 2 and the 95% confidence intervals of slope parameter values included 0. There was, therefore, no statistically significant relationship between haptoglobin and any of the life-history traits measured here. However, model 2 shows a tendency of haptoglobin to positively affect female survival rate (Fig 1B).

## Discussion

Non-breeder males having higher plasma TBARS at time t had reduced future breeding probabilities in the succeeding four years; TBARS had also a marginal negative effect on return rate, but it did not predict survival rate nor hatching and fledging success. Haptoglobin showed a non-significant tendency to positively affect female survival rate, but it was not related to any other traits.

Non-breeder males were all observed, at least once, as breeder at the time of blood sampling. Wandering albatrosses are quasi-biennial breeders, hence non-breeders were probably skipping reproduction [14]. Non-breeding individuals can represent a substantial part of animal populations [14, 22]. It is thought that nutritional and energetic costs may force birds to adopt this skipping strategy [23]. The reason for our results might also be that increased oxidative stress speeds up ageing of some reproductive traits (e.g., the hormonal control of reproductive behaviour). To some extent this explanation is supported by the fact that breeding probabilities decrease with time at a faster rate in wandering albatross males than in females [13]. However, our sample size for non-breeding females was small, which prevents us from inferring that the link between TBARS and reproductive ageing is sex-specific.

Our results also indicate that the interpretation of the biological meaning of oxidative stress should consider the individual life-cycle stage and the individual reproductive history. Breeding albatrosses have higher plasma TBARS than non-breeding albatrosses [9]; however, we only found evidence for a link between oxidative stress and demographic traits in non-breeder males. High TBARS levels in breeding individuals may indicate a transient condition, i.e., TBARS would quickly return to low basal levels when the breeding phase is finished. In contrast, TBARS levels of non-breeders (i.e., not influenced by current reproductive activity) may closely reflect a basal health status (e.g., low quality individuals; [24]), which may influence reproductive perspectives. It might also be that TBARS levels of non-breeders reflect reproductive ageing because in a previous study we found that older non-breeding individuals have higher TBARS [9]. The negative impact of TBARS on the breeding probability of non-breeder males may be relevant for the population. Non-breeders may act as a buffer in case of high adult mortality by replenishing the breeding population and so avoiding population crashes [25]. However, our results did not show a negative effect of TBARS on future hatching or fledging success, indicating that experiencing high oxidative stress during reproduction [9] might not compromise future reproductive success. These results undermine the generality of the oxidative cost/constraint of reproduction hypothesis, stating that oxidative stress experienced during reproduction should constrain future reproductive success [1]. Further studies will be necessary to ascertain whether oxidative stress markers can be used to assess or predict some demographic traits of populations [26].

Although haptoglobin was generally not related to any demographic parameters, females with higher haptoglobin at time t showed a non-significant tendency to survive longer. The haptoglobin is an acute phase protein that can limit the spread of oxidative stress across tissues [3]. We did not find evidence for a link between oxidative stress and survival. Although we found a significant but weak positive correlation between TBARS and haptoglobin, the potential positive effect of haptoglobin on female survival may be independent of its potential antioxidant role or may be related to other components of the oxidative balance that our oxidative stress marker was unable to detect. For example, haptoglobin may be important to protect against infection induced by parasites as suggested by the positive correlation we found between haptoglobin and ectoparasite density in the plumage.

Overall, we found weak to moderate relationships between physiological and demographic parameters. This may be because oxidative and inflammatory status are highly dynamic. For example, blood metrics of oxidative stress can vary across seasons and within a few hours in response to stressors [1]. Moreover, our single metrics may be unable to capture the complexity of oxidative and inflammatory status, while demographic parameters may be better reflected by the sum of multiple metrics of oxidative and inflammatory status. Another reason for our results may lie with the life-history of albatrosses. These seabirds have a slow pace of life and are very longevous, indicating that they may prioritise self-maintenance mechanisms over reproduction. Hence, albatrosses may limit OS, mitigating long-term detrimental effects on reproduction. Previous work on short-lived species does not support this explanation because individuals having high OS at time t did not always have reduced survival or reproductive perspectives [5,27]. Moreover, parents of a short-lived species have been found sacrificing current reproduction for self-maintenance under demanding conditions [28]. Similarly, work on long-lived seabirds found that oxidative stress may be associated with reduced survival or lifetime reproductive success [29–30] or may not be associated with the return rate to the colony [6]. Costs of an increased reproductive effort are not always observed [31], which could be because these costs may not be evident if resources are not limiting [32]. For example, consequences of sacrificing protection against oxidative stress for reproduction might be more likely to occur under certain environmental conditions that might limit recovery, for example poor food availability [1]. Another explanation may, however, lie with the biomarkers used to assess oxidative stress. Results suggest that there may be variation among species in the biological information provided by a same biomarker of oxidative stress [5–6, 30]. This raises the need to use multiple metrics of oxidative stress in order to examine whether damage to certain molecules (e.g., proteins) impinges on fitness traits more than that to others molecules (e.g., lipids) and whether this is dependent on the species.

In conclusion, our study shows that (i) sexually mature non-breeder males with higher levels of a marker of plasma oxidative stress (TBARS) at time t have lower future breeding probabilities in the next four years and (ii) TBARS was not related to survival rate or future hatching and fledging success. Our results also suggest (i) a potential negative effect of TBARS on return rate and (ii) a potential beneficial effect of haptoglobin on female survival. Future studies will be necessary to assess which environmental stressors influence both the oxidative and inflammatory status and how stress-induced changes in both the oxidative and inflammatory status relate to individual life-history and population viability. To this end, it will be important to use multiple markers of oxidative stress (e.g., protein carbonyls, DNA damage, markers of antioxidant defences) and multiple samples collected from the same individual over a period of time.

## Supporting Information

S1 Dataset List of data on MSMR codes, sex, TBARS and haptoglobin used for the analyses. (PDF)
