# Supplementary material for: Demographic Responses to Oxidative Stress and Inflammation in the Wandering Albatross (Diomedea exulans)
Source: PLoS One. 2015 Aug 14;10(8):e0133967. doi: 10.1371/journal.pone.0133967 (PMC4537254; doi:10.1371/journal.pone.0133967)
Supplement: S1 Dataset — (PDF) [file pone.0133967.s001.pdf]

Ring	Capture_history	Male	Female	Haptoglobin	TBARS
BS1073	30000	0	1	0,26	11,81
BS20644	34030	1	0	0,36	11,84
BS5729	44302	1	0	0,28	12,28
BS19487	43000	1	0	0,29	15,07
BS25204	44304	1	0	0,37	16,44
BS11211	34142	0	1	0,31	17,05
BS10777	30300	0	1	0,11	18,75
BS21548	44000	1	0	0,27	18,96
BS6469	44000	1	0	0,36	19,43
BS6701	43034	0	1	0,45	19,60
BS25841	44343	1	0	0,39	20,20
BS25810	20000	1	0	0,52	20,87
BS25404	30343	0	1	0,33	21,39
BS20512	33030	1	0	0,25	21,59
BS20543	30304	1	0	0,61	21,69
BS25879	40000	1	0	0,44	22,13
BS22864	30302	0	1	0,33	23,40
BS7776	43000	1	0	0,63	23,56
BS12526	30303	1	0	0,19	23,61
BS7670	30000	1	0	0,25	23,75
BS22724	13030	0	1	0,30	23,79
BS21160	40000	1	0	0,36	24,33
BS11684	30444	1	0	0,36	25,00
BS22532	40000	1	0	0,28	25,33
BS25858	30343	0	1	0,39	25,64
BS19503	34303	1	0	0,30	25,87
BS25423	30343	1	0	0,26	26,24
BS22785	30303	0	1	0,36	26,34
BS25847	43003	1	0	0,47	26,59
BS25291	30000	1	0	0,41	26,79
BS20259	30013	0	1	0,36	26,94
BS25390	30000	1	0	0,30	27,25
BS25309	30214	1	0	0,26	27,68
BS21455	30303	0	1	0,33	27,72
BS4687	10010	1	0	0,30	28,34
BS25243	40303	0	1	0,31	29,06
BS8812	43044	1	0	0,08	29,16
BS22867	30303	1	0	0,36	29,34
BS20567	30030	1	0	0,41	29,71
BS0863	30000	1	0	0,23	29,85
BS7272	44044	1	0	0,45	30,07
BS6100	40000	1	0	0,10	30,35
BS25419	30303	1	0	0,22	31,28
BS25821	34343	0	1	0,30	31,70
BS12322	43444	1	0	0,26	31,98
BS12307	30302	1	0	0,22	32,02
BS21294	24303	0	1	0,31	32,76
BS21799	31002	0	1	0,30	33,72
BS9436	14112	0	1	0,52	33,73
BS22962	10230	0	1	0,29	33,92
BS21228	30343	0	1	0,25	33,99

BS7652	12144	1	0	0,34	34,63
BS20590	13030	1	0	0,26	34,65
BS22527	30343	0	1	0,36	35,08
BS19476	13012	1	0	0,65	35,17
BS21140	30303	0	1	0,22	35,26
BS21617	13014	1	0	0,42	35,63
BS8831	34413	1	0	0,25	35,99
BS9839	30303	1	0	0,33	36,04
BS11207	30303	0	1	0,50	36,36
BS25757	13400	1	0	0,30	36,55
BS9874	30000	1	0	0,30	37,09
BS25415	30303	0	1	0,51	37,27
BS25212	14000	1	0	0,32	37,38
BS25420	30400	1	0	0,30	37,41
BS25242	13030	1	0	0,34	37,52
BS20458	20000	1	0	0,26	37,94
BS4666	30344	1	0	0,19	38,04
BS8433	40044	1	0	0,70	38,36
BS8412	40000	1	0	0,50	38,60
BS9443	30304	1	0	0,36	39,09
BS5688	13030	1	0	0,34	40,09
BS19402	30400	0	1	0,32	40,34
BS7046	33030	0	1	0,36	40,47
BS21607	30303	0	1	0,47	40,60
BS9415	34340	1	0	0,24	40,67
BS25246	30000	0	1	0,34	40,71
BS12896	30000	0	1	0,36	40,76
BS19477	30210	1	0	0,31	41,02
BS9943	13030	0	1	0,36	41,51
BS19246	34030	1	0	0,26	41,63
BS21214	13030	1	0	0,43	41,91
BS19161	30343	0	1	0,56	41,96
BS6191	30303	1	0	0,60	43,10
BS6464	40000	1	0	0,22	43,46
BS22879	30000	0	1	0,24	43,92
BS21136	30414	1	0	0,19	44,28
BS10767	30303	1	0	0,31	45,18
BS21362	30303	0	1	0,39	45,74
BS18853	30302	0	1	0,36	45,93
CF4403	40000	1	0	0,32	46,49
BS25213	22000	0	1	0,39	46,81
BS25853	34301	1	0	0,56	47,09
BS23634	30303	1	0	0,36	47,32
BS5874	44400	1	0	0,36	47,42
BS22899	30304	0	1	0,32	47,84
BS21372	30301	1	0	0,27	47,93
BS8027	30444	1	0	0,26	48,56
BS6790	40004	1	0	0,25	48,85
BS20569	30303	0	1	0,83	49,48
BS5337	34000	1	0	0,32	49,84
BS20220	30303	0	1	0,30	50,48
BS25416	12434	0	1	0,55	50,97

BS9173	30303	1	0	0,35	51,11
BS25742	44444	1	0	0,34	51,15
BS20454	30303	1	0	0,31	51,38
BS21680	13030	1	0	0,34	51,43
BS23603	30101	0	1	0,31	51,57
BS25409	30303	0	1	0,28	52,45
BS6789	40000	1	0	0,35	53,41
BS23244	13130	0	1	0,33	53,60
BS19490	12030	1	0	0,29	53,93
BS6781	44444	1	0	0,97	54,91
CF4401	40002	0	1	0,35	55,24
BS0957	34301	0	1	0,26	55,85
BS25203	32303	1	0	0,36	56,42
BS21644	30303	0	1	0,48	57,76
BS6104	20000	1	0	0,21	58,22
BS20575	30000	1	0	0,25	59,43
BS9450	30010	0	1	0,29	61,78
BS6098	40000	1	0	0,32	63,10
BS18938	30000	1	0	0,37	63,86
BS20532	30301	0	1	1,00	64,23
BS6570	44444	1	0	0,36	64,51
BS8046	30304	1	0	0,10	65,03
BS25210	30303	0	1	0,35	65,05
BS21258	13030	0	1	0,54	66,91
BS6244	40040	1	0	0,45	70,95
BS4774	40440	1	0	0,26	83,36
BS11240	43030	0	1	0,87	86,32
BS22863	30313	0	1	0,32	95,00
BS25198	30302	1	0	0,36	101,40
BS19840	30303	0	1	0,36	102,22
BS10875	23030	1	0	0,28	109,97
BS21453	30000	0	1	0,40	111,33
BS23622	11211	0	1	0,62	41,40
BS19808	30300	1	0	0,34	41,40
BS25216	30333	0	1	0,37	41,40
BS5690	41441	1	0	0,51	41,40
BS10342	40000	1	0	0,42	41,40
BS21144	30003	0	1	0,42	41,40
BS1169	40004	1	0	0,34	41,40
BS20583	30302	1	0	0,40	41,40
BS20283	30304	1	0	0,42	41,40
